# Primary Evaluation of Three-Dimensional Printing-Guided Endodontics in the Dog Maxillary

**DOI:** 10.3390/vetsci12070665

**Published:** 2025-07-14

**Authors:** Chengli Zheng, Xiaoxuan Pan, Jiahui Peng, Xiaoxiao Zhou, Xin Shi, Liuqing Yang, Yan Luo, Haifeng Liu, Zhijun Zhong, Guangneng Peng, Min Yang, Ming Zhang, Ziyao Zhou

**Affiliations:** 1 College of Veterinary Medicine, Sichuan Agricultural University, Chengdu 611130, China; 2 Sichuan Institute of Musk Deer Breeding, Sichuan Institute for Drug Control, Chengdu 611830, China; 3 Chengdu Center for Animal Disease Prevention and Control, Chengdu 610041, China; 4 Pet Nutrition and Health Research Center, Chengdu Agricultural College, Chengdu 611130, China; 5 College of Animal Science and Technology, Sichuan Agricultural University, Chengdu 611130, China

**Keywords:** 3D printing, guided endodontics, maxillary teeth, finite element analysis, veterinary dentistry, root canal treatment

## Abstract

This study aims to evaluate the feasibility and accuracy of 3D printing-guided endodontics in the maxillary teeth of dogs. CT data from a Beagle dog were processed to create a 3D model of the maxilla, and virtual root canal pathways were established using SOLIDWORKS software (version 29.0.0.5028). Guided endodontic templates were 3D printed and tested in vitro on 20 maxillary teeth (excluding the third molars), with 36 root canals treated using both guided and conventional methods. Results indicated that 3D printing-guided endodontics provided accurate root canal pathways, with minimal deviations in length (average 3.08 ± 1.75%) and angular alignment (average 2.06° ± 0.5°) compared to conventional methods. This research represents a significant step forward in the application of 3D printing technology in veterinary endodontics, offering a promising alternative to traditional methods for treating complex dental conditions in dogs.

## Simple Summary

Our previous research demonstrated that 3D printing in guided endodontics provides accurate positioning, direction, and length in the mandible region of dogs. However, it may have relative errors in some special teeth, particularly in the case of teeth with multi-roots. This study presents an advanced approach to veterinary endodontic treatments in the maxilla of Beagle dogs, particularly regarding the three-rooted fourth premolar. This study highlights the potential of 3D printing-guided endodontics to improve the precision and success rate of root canal treatments in veterinary dentistry. Limitations include breed-specific anatomical variations and the need for further clinical validation.

## 1. Introduction

Our previous research demonstrated that 3D printing in guided endodontics provides accurate positioning, direction, and length in the mandible region of dogs [1]. The benefits of guided endodontics are as follows: (1) finite element analysis (FEA), when merged with customized computed tomography (CT) data, can yield highly accurate representations of individual anatomical conditions and precise three-dimensional information [2,3,4]; (2) the ability to generate personalized implants, which can decrease the fabrication time and expenses [5]; (3) operative planning might considerably increase the success rate of surgical interventions [6]. However, this situation may have relative errors in some special teeth, particularly in the case of multi-rooted teeth [7]. In the maxilla of dogs, notably, the maxillary fourth premolar and first molar may present a challenge, as the mesiopalatal and buccal roots share a common canal open [8,9]. These complexities pose significant challenges for guided endodontics.

The aim of this study is to address these challenges by simulating dental pulp procedures through the interpretation of CT data, the establishment of mathematical models, precision 3D printing for endodontics, and the assessment of root canal pathways via radiographic analysis. Our study introduces an innovative method to overcome the difficulties encountered in veterinary dental pulp procedures in the maxilla of dogs, particularly regarding the three-rooted fourth premolar.

## 2. Materials and Methods

### 2.1. Creation of a 3D Model of the Beagle Maxillary

**Figure 1:**
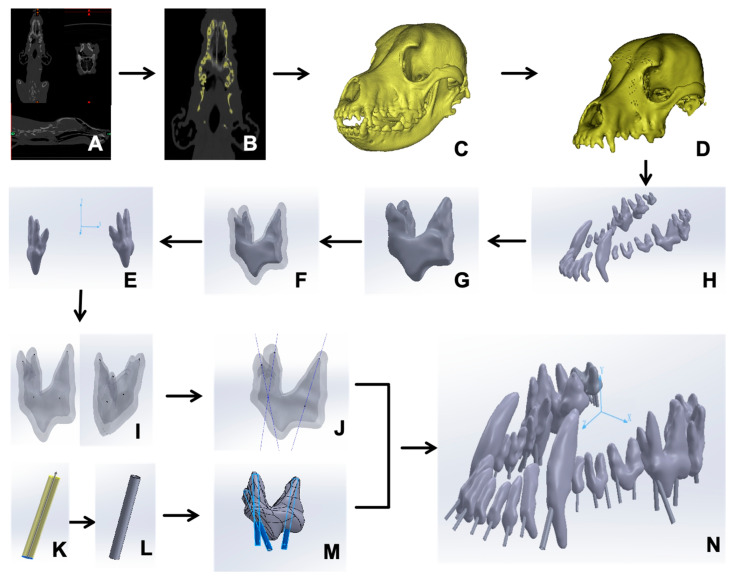
Scheme of the Beagle maxillary 3D model construction. (**A**) CT scanned data. (**B**) Head skull image reconstructed. (**C**) Preliminary 3D head skull model generated. (**D**) Separation of the maxillary skull. (**E**) Teeth segmented. (**F**) Individual tooth segmented. (**G**) Dental pulp reconstructed. (**H**) Dental coordinate reduction. (**I**) Determination of the center and apex of the root canal orifice of the tooth. (**J**) Identification of the root canal orientation. (**K**) Virtual root canal model. (**L**) Accurate root canal model. (**M**) The guided model fit to the pulp. (**N**) All maxillary teeth assembled and positioned.

A Beagle dog without head trauma, scanned in our previous study, was investigated through computer modeling [1]. The dog was provided by the Sichuan Science and Technology Resources Sharing Platform for Beagle Dog Breeding and Experimental Technology Service. The CT scans (Figure 1A) were initially loaded into Mimics Medical (Materialise Co., Ltd., Leuven, Belgium, Version 22.0.306), which is a universal medical computer imaging software program in DICOM format (Figure 1B). A 3D mesh model of the Beagle maxillary was generated using the “New Mask” function. (Figure 1C).

Next, the stereolithography file was imported into Geomagic Wrap (Geomagic Co., Ltd., Research Triangle Park, NC, USA, version 2021.0.0.3008) for shell smoothing via the “Feature Removal” and “Hole Filling” tools. Subsequently, the “Precise Surface” options were harnessed to finely adjust the surface, permitting targeted modifications and eliminating noise, so that a refined 3D model of the Beagle maxillary bone (Figure 1D) was created. Eventually, the maxillary graphics obtained were saved in STP format and introduced into SOLIDWORKS (Dassault Systèmes S.A. Co., Ltd., Vélizy-Villacoublay, France, version 29.0.0.5028) for the purpose of materialization.

### 2.2. Extraction of Maxillary Teeth

In SOLIDWORKS, 20 maxillary teeth were identified and virtually extracted using the “Region Growing” function (Figure 1E). Each tooth was reconstructed using the “3D Editing” and “Boolean Operations” functions to eliminate artifacts, resulting in a refined surface polygon for each tooth (Figure 1F) [10]. The pulp chamber and dentin were separately reconstructed (Figure 1G) [10]. Within SOLIDWORKS, the “Coordinate System” feature was neatly employed to generate a 3D coordinate system made up of the XYZ axes, after which they were exported in STL format (Figure 1H).

### 2.3. Formulation of Root Canal Pathways and Guiding Templates

In SOLIDWORKS, the origin point (0, 0, 0) was designated as the anatomical midpoint of the skull. The “Reference Geometry” feature was used to ascertain the position of the peak point (x1, y1, z1) and of the central point of each dental root canal orifice (x2, y2, z2) for every tooth (Figure 1I). In theory, a direct line should link these two points within the three-dimensional space. Subsequently, the “Curve Through XYZ Points” feature was utilized to determine the linear pathway of the root canal for the maxillary tooth (Figure 1J).

The root canal guide template, with a thickness of 1 mm, was created from the line connecting the root canal to the tooth surface, serving as the guide for the procedure. The maximum diameter of the root canal was measured and the corresponding three-dimensional orifice was produced in a guided model (Figure 1K,L). The guide hole extends to the incisal edge to facilitate surgical maneuvering (Figure 1M). To safeguard the preciseness of each tooth’s positioning, the entire skull model was likewise incorporated (Figure 1N). The resulting model was exported as an STL file for 3D printing.

### 2.4. Root Canal Experiment in Vitro

In vitro root canal experiments were conducted on two distinct Beagle skull models provided by the Sichuan Science and Technology Resources Sharing Platform for Beagle Dog Breeding and Experimental Technology Service. A total of 36 root canals were performed on maxillary teeth, totaling 20 teeth (including 6 incisors, 2 canines, 8 premolars, and 4 molars), using guided endodontic and conventional treatment methods, respectively. The first Beagle maxillary model was utilized for guided endodontic treatment, with initially printed templates for each tooth according to the manufacturer’s instructions (SLA550Li, material: photosensitive resin; Ultrust Imaging (Chengdu) Center Co., Ltd., Chengdu, China). The fit of the template to the printed corresponding tooth was verified for accuracy and reproducibility.

This study focused on evaluating the relative error in root canal length measurement in two methods. Therefore, the method did not involve complete root canal treatment. The specific procedures were described according to the guidelines for reporting case reports in endodontics, specifically from the PRICE explanation and elaboration document [11] as follows: A high-speed dental drill (SKU: VetPro 1000, from Midmark, Versailles, OH, USA) was entered into the template to create an access cavity. The pulp chamber was irrigated with sodium hypochlorite to remove residual pulp tissue. Moisture control was achieved using sterile paper points. Standardized endodontic K-files (ISO 3630-1 [12] specifications) (yellow: diameter 0.20 mm, length 21 mm, and taper 0.02; red: diameter 0.25 mm, length 21 mm, and taper 0.02; blue: diameter 0.30 mm, length 21 mm, and taper 0.02) were employed for canal exploration and length determination using a dental X-ray (Ningbo Juxing Peteeth Medical Smart Technology Co., Ltd., Ningbo, China).

Conventional endodontic treatment was performed on another Beagle maxilla, which followed the same procedure as the guided endodontics treatment but without employing the template. In both methods, the root canal length was measured from the center point of the root canal opening to the bottom of the pulp chamber. A comparative assessment of angular deviation was also performed to evaluate the accuracy of the guided root canal treatment versus the conventional techniques.

### 2.5. Statistical Analysis

Statistical assessments were carried out on the measured root canal lengths and angular discrepancies between the two methods. The comparison of data distributions was executed using the Student’s t-test via SPSS v27 (IBM, New York, NY, USA). A *p*-value below 0.05 was regarded as statistically significant.

## 3. Results

### 3.1. Computer-Aided Design of the Root Canal Guided Lines

Based on a three-dimensional coordinate system, a total of 20 guided endodontic templates were designed for 20 maxillary teeth. The coordinates of the apex of the pulp chamber (x1, y1, z1) and the central point of each dental root canal orifice (x2, y2, z2) for each maxillary tooth are listed in Table 1. Taking the left fourth premolar (208) as an illustration, the diameters of the two pulp crowns were 4.70 mm and 4.27 mm, respectively. The coordinates of the mesial palatine root in 3D space were measured as (19.84, 5.76, 1.27) and (22.58, −17.97, 2.63). The coordinates of the mesial buccal root were (15.53, 0.21, 1.64) and (21.37, −8.46, 2.57). The coordinates of the distal root were (23.00, 3.75, −9.03) and (24.10, −9.05, −5.70). Three root canal guide lines can be generated by connecting the lines based on the coordinates.

### 3.2. Three-Dimensional-Printed Maxillary and Guided Endodontic Templates

**Figure 2:**
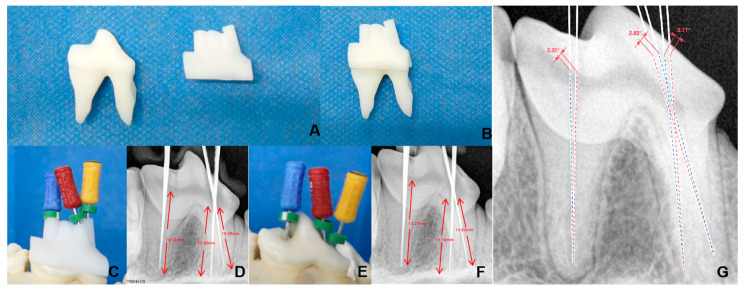
Comparison of treatments by guided endodontics and conventional root canal methods. (**A**) The 3D-printed left fourth premolar tooth and its template. (**B**) Combination of 3D-printed root canal template and the printed tooth for verifying the fits. (**C**) Guided root canal treatment method. (**D**) Radiograph-based guided endodontics. (**E**) Removal of guided molding. (**F**) Radiograph-based conventional root canal method. (**G**) Comparison of root canal angles between the guided (blue line) and conventional (red line) root canal treatment methods.

Three-dimensional-guided endodontic templates were printed based on the created pattern, demonstrating a precise fit between the template and the printed teeth (Figure 2A). Specifically, the left fourth premolar (208) measured 15.35 mm in length, 6.69 mm in width, and 21.30 mm in height. The template dimensions were 15.21 mm × 8.62 mm × 11.29 mm, which were fully compatible with the printed tooth (Figure 2B). The root canal pathway was also fitted to the pulp chamber by using the Beagle maxillary model (Figure 2C,D).

### 3.3. Comparison of 3D Printing-Guided Endodontics and Conventional Root Canal Methods

The root canal treatment experiments were performed and evaluated using 36 maxillary pulp cavities of Beagle dogs. The left fourth premolar (208), which possesses a complex root, was selected for X-ray scanning, and the tip of the gum was observed to be straight to the apex without bending, damage, or excessive length. The length of the mesiopalatal root and mesiobuccal root canal was 10.49 mm and 14.26 mm, respectively; meanwhile, the distal root canal measured 14.02 mm (Figure 2D). In contrast, the length of the mesiopalatal and mesiobuccal root canal using the conventional method was 11.19 mm and 14.83 mm, respectively, and the distal root canal measured 13.27 mm (Figure 2F).

The angular deviations of the root canal pathway between the two methods were 2.77° (mesiopalatal root), 2.83° (mesiobuccal root), and 2.20° (distal root), respectively (Figure 2G). To enhance clarity and readability, the root canal orientations of the guided sleeve method and the conventional method were plotted separately in Figure 2G.

Of the 36 root canals, the relative deviations between the guided endodontics and conventional methods ranged from 0.16% (106 distal root) to 6.67% (208 mesiobuccal root), with an average deviation of 3.08% ± 1.75%. The mean angular deviation was 2.06° ± 0.5°, ranging from 1.17° (201 root) to 2.41° (107 mesial root) (Table 2).

Of the maxillary teeth, the premolars exhibited the most considerable variation in root canal length, amounting to 3.55% ± 2.03% of the standard deviation. This variation was significantly higher than that of the incisors (1.44% ± 0.66%, *p* = 0.022) but was similar to that of the molars (3.60% ± 1.03%, *p* = 0.932). However, there was no significant difference in angular deviation among the teeth, with the incisors measuring 1.45° ± 0.35° (*p* = 0.6778 for incisors vs. premolars), premolars 2.35° ± 0.39° (*p* = 0.9825 for premolars vs. molars), and molars 2.09° ± 0.32° (*p* = 0.7806 for incisors vs. molars), respectively.

## 4. Discussion

Currently, endodontic treatment in veterinary dentistry mostly relies on the operator’s clinical knowledge base and expertise. Several potential causes of failure during pulp access include the following: (1) deviations during entry in the root canal treatment, such as incorrect angles of access to the pulp chamber, incomplete exposure of the chamber, inadequate exposure of the canal orifice, perforations in the pulp chamber floor or canal walls, overlooked canals, or incorrect interpretation of canal anomalies (e.g., S-shaped canal configurations) [13,14]; (2) deviations in canal preparation, which may involve excessive canal enlargement, ledge formation, instrument separation, or incomplete cleaning of the canal. For instance, Lee et al. [15] evaluated the outcomes of endodontic treatments in 204 dogs, noting that multi-root teeth with more complex canal morphologies may result in higher fail rates. Therefore, developing an adjunctive endodontic method with computer-aided design contributes to diminishing the likelihood of complications arising and to enhancing the overall outcome for patients [16,17].

Laboratory and in vitro investigations have demonstrated considerable precision in aligning actual canal trajectories with pre-determined pathways through the application of guided procedures [18,19]. Our previous research indicated that guiding root canal treatment in dogs’ mandibular canals is comparable to traditional endodontic techniques employed by dental specialists [1]. This method enables veterinarians to formulate feasible plans for actual clinical procedures, aiming for optimal positioning and angles, ultimately minimizing the removal of valuable dental structures. However, certain complex canal situations remain unknown, particularly in the maxillary fourth premolars, which are among the most commonly fractured and treated teeth in dogs [20]. In this study, our findings suggest that utilizing a 3D-printed guide for root canal treatment can be quite suitable for maxillary teeth, including the frequently encountered complex fourth premolars. However, compared to single-rooted teeth (such as incisors and canines), the use of guided endodontics in multi-rooted teeth indeed results in greater discrepancies. This outcome may be attributed to the more complex fit of the guiding template with the canals, making it easier to introduce angulation errors during clinical procedures. Consequently, it is highly recommended to calibrate the coordinates and dimensions in SOLIDWORKS software, before printing 3D root canal templates, to avoid mismatches between the template and canals after final assembly. This is particularly evident when two or more canals share a common open pulp chamber, significantly affecting the procedural difficulty [21]. However, this issue is a result of the anatomical structure of the teeth rather than our method. In traditional approaches, endodontic treatment of these multi-rooted teeth is also complex and has a high failure rate [22]. We hope our method might serve as a better solution for these intricate canal procedures.

Nonetheless, this study has some inherent limitations, including the following: (1) the guided pulp templates and measured parameters are individualized for Beagles, which means that they may not be applicable to other breeds; (2) even within the same breed, variations in size, sex, age, and dental structure can lead to significant differences in root canal templates produced from dogs of the same breed; (3) the addition of CT scans and the production of 3D guiding templates may extend operation and anesthesia times, which can be particularly impactful for complex dental cases and older animals, posing significant challenges to practitioners’ skills and experience [23]. In summary, FEA-guided endodontic treatment represents a safe and direct approach, especially considering the further developmental potential of 3D printing technology [24,25]. However, FEA-guided endodontic treatment may require additional equipment and increase the cost of veterinary dentistry. Additional clinical research might be needed to fully determine the clinical advantages of these new guiding techniques.

## Figures and Tables

**Table 1:** Coordinates of the apex point (x1, y1, z1) and the central point of the dental root canal orifice (x2, y2, z2) for each tooth.

Tooth Number	Root Position	Apex Point (x_1_, y_1_, z_1_)	Central Point of the Dental Root Canal Orifice (x_2_, y_2_, z_2_)	Tooth Number	Root Position	Apex Point (x_1_, y_1_, z_1_)	Central Point of the Dental Root Canal Orifice (x_2_, y_2_, z_2_)
101	/	(−1.84, 5.78, 51.91)	(−0.88, 1.03, 60.62)	201	/	(3.19, 5.84, 51.10)	(4.01, 1.42, 59.73)
102	/	(−4.55, 6.43, 49.65)	(−5.85, 1.17, 59.41)	202	/	(5.74, 7.03, 48.82)	(8.81, 1.21, 58.28)
103	/	(−6.89, 7.03, 46.31)	(−10.34, 0.93, 55.93)	203	/	(6.85, 7.40, 43.00)	(11.84, 2.32, 54.22)
104	/	(−12.30, 13.23, 23.33)	(−13.64, −0.42, 44.52)	204	/	(9.42, 14.41, 25.21)	(13.98, 1.30, 42.73)
105	/	(−13.72, 3.27, 29.12)	(−14.22, −2.82, 33.54)	205	/	(11.87, 3.96, 27.36)	(14.44, −1.42, 31.66)
106	mesial	(−14.52, 1.48, 24.99)	(−14.58, −5.28, 26.86)	206	mesial	(12.36, 3.40, 23.84)	(14.84, −2.93, 25.23)
	distal	(−14.47, 0.42, 19.42)	(−16.16, −5.03, 21.69)		distal	(12.97, 0.95, 18.55)	(15.86, −3.76, 20.54)
107	mesial	(−15.72, 0.36, 13.91)	(−17.09, −6.14, 14.57)	207	mesial	(13.50, 1.32, 13.13)	(16.15, −3.96, 15.00)
	distal	(−18.44, −0.84, 8.17)	(−20.25, −7.27, 9.93)		distal	(16.62, 0.40, 6.68)	(19.04, −5.43, 9.79)
108	mesiobuccal	(−17.27, −0.41, 3.09)	(−22.41, −10.18, 3.83)	208	mesiobuccal	(15.53, 0.21, 1.64)	(21.37, −8.46, 2.57)
	mesiopalatal	(−23.07, 3.62, 3.06)	(−22.41, −10.18, 3.83)		mesiopalatal	(19.84, 5.76, 1.27)	(22.58, −17.97, 2.63)
	distal	(−26.45, 1.46, −6.39)	(−25.10, −10.69, −3.45)		distal	(23.00, 3.75, −9.03)	(24.10, −9.05, −5.70)
109	mesiobuccal	(−27.84, −3.89, 10.04)	(−25.47, −13.04, −11.20)	209	mesiobuccal	(16.34, 0.19, −15.28)	(17.62, −8.05, −14.04)
	mesiopalatal	(−20.62, 2.96, −14.25)	(−19.85, −9.75, −12.74)		mesiopalatal	(24.37, −0.17, −18.00)	(22.86, −8.32, −16.81)
	distobuccal	(−28.12, −3.51, −16.38)	(−25.14, −12.14, −14.89)		distobuccal	(24.83, −1.94, −12.00)	(24.54, −9.57, −13.16)
110	mesiobuccal	(−24.82, 5.03, −19.16)	(−22.23, −8.93, −20.12)	210	mesiobuccal	(16.49, −1.93, −24.18)	(16.80, −6.21, −24.17)
	mesiopalatal	(−18.22, −2.86, 19.99)	(−17.48, −7.54, −20.01)		mesiopalatal	(13.21, −1.88, −21.51)	(13.84, −6.00, −21.97)
	distobuccal	(−21.29, 4.10, −22.51)	(−19.96, −8.32, −21.95)		distobuccal	(20.78, −2.00, −20.96)	(18.96, −7.39, −21.85)

**Table 2:** Length measurement of each root canal using guided and conventional techniques.

Tooth Number	Root Position	Guided Endodontics/mm	Classic Method/mm	Deviation Rate	Angular Deviation	Tooth Number	Root Position	Guided Endodontics/mm	Classic Method/mm	Deviation Rate	Angular Deviation
101	/	9.97	9.87	1.00%	2.38°	201	/	9.73	9.5	2.36%	1.17°
102	/	11.16	11.34	1.61%	1.65°	202	/	11.52	11.75	2.00%	2.03°
103	/	11.9	11.98	0.67%	2.08°	203	/	13.29	13.42	0.98%	1.41°
104	/	24.01	24.46	1.87%	1.28°	204	/	23.92	23.82	0.42%	1.34°
105	/	7.55	7.47	1.06%	1.81°	205	/	7.36	7.19	2.31%	2.24°
106	mesial	7.12	7.41	4.07%	2.17°	206	mesial	6.93	6.85	1.15%	1.31°
	distal	6.14	6.13	0.16%	1.45°		distal	5.88	6.13	4.25%	1.64°
107	mesial	6.67	6.54	1.95%	2.41°	207	mesial	6.20	6.56	5.81%	2.4°
	distal	6.90	6.84	0.87%	1.77°		distal	7.04	6.79	3.55%	1.68°
108	mesiobuccal	11.06	11.67	5.52%	2.21°	208	mesiobuccal	10.49	11.19	6.67%	1.83°
	mesiopalatal	13.63	14.31	4.99%	2.27°		mesiopalatal	14.26	14.83	4.00%	1.77°
	distal	12.54	11.91	5.02%	1.72°		distal	14.02	13.27	5.35%	2.2°
109	mesiobuccal	9.53	9.17	3.78%	1.61°	209	mesiobuccal	7.73	7.61	1.55%	1.41°
	palatal	6.99	7.29	4.29%	2.25°		palatal	8.65	8.43	2.54%	1.98°
	distobuccal	9.24	9.57	3.57%	2.07°		distobuccal	8.38	8.75	4.42%	1.36°
110	mesiobuccal	4.77	4.97	4.19%	1.75°	210	mesiobuccal	5.55	5.39	2.88%	2.17°
	palatal	4.94	5.11	3.44%	2.18°		palatal	4.29	4.41	2.80%	1.87°
	distobuccal	5.73	5.47	4.54%	1.94°		distobuccal	4.40	4.63	5.23%	2.28°

## Data Availability

The original contributions presented in this study are included in the article. Further inquiries can be directed to the corresponding authors.
